# The influence of chronic renal insufficiency on multi-therapeutic modalities for breast cancer: a single-center experience

**DOI:** 10.1007/s12282-023-01530-w

**Published:** 2023-12-27

**Authors:** Yi-Wen Hong, I-Ming Kuo, Wen-Ling Kuo, Chi-Chang Yu, Shih-Che Shen, Hsiu-Pei Tsai, Chia-Hui Chu, Hui-Yu Ho, Yung-Feng Lo, Shin-Cheh Chen, Yung-Chang Lin, Chih-Ying Chien, Hsu-Huan Chou

**Affiliations:** 1 Division of General Surgery, Department of Surgery, New Taipei Municipal TuCheng Hospital, No. 6, Sec. 2, Jincheng Rd., Tucheng Dist., New Taipei City, 236 Taiwan; 2 Division of General Surgery, Department of Surgery, Chang Gung Memorial Hospital, Chang Gung University, No. 5, Fuxing St., Guishan Dist., Taoyuan City, 333 Taiwan; 3 Division of Medical Oncology, Department of Internal Medicine, Chang Gung Memorial Hospital, Linkou, Taiwan; 4 Division of General Surgery, Department of Surgery, Chang Gung Memorial Hospital, Keelung, Taiwan

**Keywords:** Breast cancer, Chronic kidney disease, End-stage renal disease, Chemotherapy, Prognosis, Propensity score matching

## Abstract

**Background:**

Due to the presence of other comorbidities and multi-therapeutic modalities in breast cancer, renally cleared chemotherapeutic regimens may cause nephrotoxicity. The aim of this retrospective study is to compare the chemotherapy types and outcomes in breast cancer patients with or without chronic renal disease.

**Patients and Methods:**

We retrospectively enrolled 62 female patients with breast cancer and underlying late stages (stage 3b, 4, and 5) of chronic kidney disease (CKD) treated from 2000 to 2017. They were propensity score-matched 1:1 with patients in our database with breast cancer and normal renal function (total *n* = 124).

**Results:**

The main subtype of breast cancer was luminal A and relatively few patients with renal impairment received chemotherapy and anti-Her-2 treatment. The breast cancer patients with late-stage CKD had a slightly higher recurrent rate, especially at the locally advanced stage. The 5-year overall survival was 90.1 and 71.2% for patients without and with late-stage CKD, but the breast cancer-related mortality rate was 88.9 and 24.1%, respectively. In multivariate analyses, dose-reduced chemotherapy was an independent negative predictor of 5-year recurrence-free survival and late-stage CKD was associated with lower 5-year overall survival rate.

**Conclusions:**

Breast cancer patients with late-stage CKD may receive insufficient therapeutic modalities. Although the recurrence-free survival rate did not differ significantly by the status of CKD, patients with breast cancer and late-stage CKD had shorter overall survival time but a lower breast cancer-related mortality rate, indicated that the mortality was related to underlying disease.

## Introduction

According to the GLOBOCAN 2020 estimates of cancer incidence and mortality produced by the International Agency for Research on Cancer [1], breast cancer is the most common malignancy affecting people worldwide, with female breast cancer surpassing lung cancer in 2020. Based on multi-therapeutic modalities, invasive breast cancer is the fifth leading cause of cancer mortality worldwide. Chronic kidney disease (CKD) directly impacts the morbidity and mortality of therapeutic program for all kinds of cancers; Taiwan has one of the world’s highest incidence and prevalence rates of CKD [2, 3]. Systemic reviews [4–7] indicate that regular screening mammography can increase the early detection of breast pre-malignancies or malignancies and reduce the risk of late-stage breast cancer, decreasing breast cancer-specific morbidity and mortality. Thus, the ten-year breast cancer-specific survival rate is reported more than 70% in recent studies [8, 9].

However, patients with CKD are at a higher risk of cancer overall, due to irregular screening programs [10], which may result in a higher breast cancer-specific incidence in this population [11]. Patients with advanced CKD often also have hyperparathyroidism, with resulting calcifications throughout their soft tissues, which increases the risk of delay in screening for breast cancer [12]. Chemotherapeutic regimens are still crucial in treating most breast cancers and are suggested by the National Comprehensive Cancer Network (NCCN) or European Society for Medical Oncology. Most of chemotherapeutic regimens are eliminated by kidneys and hepatobiliary system, which may cause nephrotoxicity and require drugs dosage adjustment. Turin et al. reported that women aged 40 years had a life expectancy of 34.6, 28.7, 16.5, and 9.1 years at CKD stage 2, 3a, 3b, and 4, respectively [13]. However, inappropriate dosage may result in worse overall survival (OS) [14, 15]. Because the life expectancy of patients with late-stage CKD might not be far different from those with breast cancer, better information is needed to refine chemotherapies in patients with breast cancer and chronic renal disease, to maximize outcomes. Our purpose is to clarify the influence of various chemotherapeutic regimens on the prognosis of patients with breast cancer and chronic renal insufficiency, to increase the precision of therapeutic guidance.

## Materials and methods

### Definition and selection criteria

The stages of CKD were classified from 1 to 5 based on the definition of Kidney Disease: Improving Global Outcomes (KDIGO) guidelines [16], and the late-stage CKD was defined as an estimated glomerular filtration rate (eGFR) less than 45 ml/min/1.73 m^2^ (CKD stage 3b, 4, and 5). A total of 62 female patients diagnosed as having breast cancer with underlying late-stage CKD treated at Chang Gung Memorial Hospital from 2000 to 2017 were retrospectively recruited. Patients with CKD were randomly matched by propensity score matching from the same period in a 1:1 ratio with patients of breast cancer and normal renal function from our database, controlling for age, stage at diagnosis, and subtypes. The pathologic stage ranged from pT1N0 to pT4N3. Those with distant metastatic status at initial diagnosis, incomplete data, or male sex were excluded. Those with diminished chemotherapeutic dose amounts or cycles of regimens were defined as dose reduction. To quantify the possible impact of dose reduction, the relative dose intensity (RDI) [17] was calculated, too. The RDI of chemotherapy is a measure of the actual dose of chemotherapy drugs received by a patient compared to the planned or standard dose. For calculating the RDI of multiple chemotherapy regimens, each chemotherapy drug or regimen should be measured separately using the formula of: RDI (%) = (Total dose received/Total planned dose) × 100. If the patient is receiving multiple chemotherapy drugs simultaneously or different chemotherapy regimens sequentially (one after the other), the overall RDI (%) is calculated as (Total combined dose received/Total combined planned dose) × 100.

### Data collection and statistical analysis

Information on demographics, characteristics of the primary tumor and axillary lymph nodes, tumor grade, pathological data, surgical details, therapeutic modalities, dosage of chemotherapeutic regimens, adjuvant therapy, period of follow-up, and the reasons for morbidity and mortality was collected from medical records and telephone interview. The American Joint Committee on Cancer 8th Edition Anatomic Staging System was used for clinical and pathologic staging. The choices of chemotherapeutic regimens were based on the NCCN guideline. In addition to the above data, we also analyzed the adjustment of dosage or cycles by patient’s renal status.

Continuous data were presented as median with interquartile range, and categorical variables were presented as percentages. The Mann–Whitney *U* test and Pearson’s χ^2^ test or Fisher’s exact test were used to identify statistically significant differences between the CKD and non-CKD groups. Outcome measures included recurrence-free survival (RFS) and OS after mastectomy and were estimated using the Kaplan–Meier method; any significant difference between subgroups (detected by univariate analysis) was compared using the log-rank test. Multivariate analysis was conducted using the Cox regression model. A *P* value < 0.05 was considered statistically significant. All statistical analyses were performed using the statistical software SPSS version 25.0 (IBM Corp., Armonk, NY, USA).

## Results

### Study characteristics

In total, 124 breast cancer patients with or without late-stage CKD were retrospectively reviewed and compared. The demographic and clinical characteristics of patients with or without CKD are listed in Table 1. The median age of breast cancer patients was 59.5 years in both groups. Modified radical mastectomy was performed in 75.8% of those in the CKD group and 77.4% in non-CKD group. The major pathology was invasive ductal carcinoma: 85.5% in the CKD group and 87.1% in the non-CKD group. The breast cancer grading, done using the modified Bloom–Richardson grading system, found that 61.3% of those in the CKD group and 72.5% in the non-CKD group were in the main high-risk group. In the two groups, the cancer staging was 38.7% at stage I, 32.2% at stage II, and 22.6% at stage III. The primary subtype of breast cancer was luminal A (58.1%), followed by triple-negative breast cancer (19.3%), luminal B (11.3%), and Her-2-enriched (11.3%). In the non-CKD group, 67.7% of patients received chemotherapy, compared to 32.3% in the CKD group. The percent receiving chemotherapeutic regimens with dose reduction was 55% in the CKD group and 14.3% in the non-CKD group. Finally, 4.8% of patients in the CKD group and 11.3% in the non-CKD group received anti-Her-2 therapy.

**Table 1:** Clinical characteristics and demographics of breast cancer patients with or without CKD

	Non-CKD	CKD	*P* value
	*N* = 62	*N* = 62	
Age (years), median (IQR)	59.5 (53.2–64.5)	59.5 (53.0–64.5)	0.974
Type of mastectomy, *n* (%)			0.832
Simple or modified radical	48 (77.4)	47 (75.8)	
Partial	14 (22.6)	15 (24.2)	
Histology, *n* (%)			1.000
Ductal carcinoma in situ	4 (6.5)	4 (6.5)	
Invasive ductal carcinoma	54 (87.1)	53 (85.5)	
Invasive lobular carcinoma	3 (4.8)	3 (4.8)	
Others	1 (1.6)	2 (3.2)	
Grade, *n* (%)			0.184
1	15 (24.2)	17 (27.4)	
2	26 (41.9)	25 (40.3)	
3	19 (30.6)	13 (21.0)	
Stage, *n* (%)			1.000
0	4 (6.5)	4 (6.5)	
I	24 (38.7)	24 (38.7)	
II	20 (32.2)	20 (32.2)	
III	14 (22.6)	14 (22.6)	
Subtype, *n* (%)			1.000
Luminal A	36 (58.1)	36 (58.1)	
Luminal B	7 (11.3)	7 (11.3)	
Her-2-enriched	7 (11.3)	7 (11.3)	
Triple-negative	12 (19.3)	12 (19.3)	
Chemotherapy, *n* (%)	42 (67.7)	20 (32.3)	< 0.0005
Dose reduction	6 (14.3)	11 (55.0)	0.002
Average RDI (%)	91.0	77.1	0.036
Hormone therapy, *n* (%)	41 (66.1)	41 (66.1)	1.000
Radiotherapy, *n* (%)	21 (33.9)	23 (37.1)	0.707
Her-2-positive^a^, *n* (%)	12 (19.4)	14 (22.6)	0.659
Anti-Her-2 therapy, *n* (%)	7 (11.3)	3 (4.8)	0.323

*CKD* chronic kidney disease, *IQR* interquartile range, *RDI* relative dose intensity

^a^Her-2-positive: Her-2 (3+) or Her-2 (2+ with positive FISH (fluorescent in situ hybridization))

Among a total of 62 CKD patients, 15 received either continuous ambulatory peritoneal dialysis (CAPD) or hemodialysis (HD). The reasons for dialysis were as follows: diabetes mellitus-related (five patients), hypertension-related (two patients), glomerulonephritis-related (two patients), obstructive uropathy-related (one patient), metabolic disease-related (one patient with high triglycerides), long-term herb-related (one patient using Chinese herbs), and unknown reasons (three patients). After completing breast cancer treatment, the status of CKD progressed in two patients, which resulted in requirement of dialysis: one experienced acute hepatic failure and renal failure due to reactivation of hepatitis B, while the other developed neutropenia-related sepsis, which led to heart failure (ejection fraction decreased from 60 to 20%) and acute-on-chronic renal failure.

### Chemotherapeutic regimens, dosage adjustment, and relative dose intensity (RDI)

Table 2, Table 3, and Table 4 list the chemotherapeutic regimens received. In the CKD group, 32.3% of the patients received chemotherapy, and about one-half of the patients in the non-CKD group. Meanwhile, the rate for adjustment of dosage, regimen, or cycles was as high as 55% in the CKD group, higher than in the non-CKD group. The rate of receiving chemotherapy for stage I, II, and III breast cancer was 8.3, 35, and 78.6% in the CKD group, and 62.5, 65, and 100% in the non-CKD group, respectively. The average RDI in CKD group was 77.1 and 91.0% in non-CKD group (*P* = 0.036). Besides, the RDIs of those with dose-reduced chemotherapy were calculated and listed in the Table 3 and Table 4.

**Table 2:** The standard guidelines of used chemotherapy regimens in the cohorts

Chemotherapy regimens	Standard dose	Number of cycles	Length of cycle (day)
CEF			
Cyclophosphamide (C)	500 mg/m^2^		
Epirubicin (E)	90 mg/m^2^	4	21
5-fluorouracil (F)	500 mg/m^2^		
AC			
Adriamycin (Doxorubicin) (A)	60 mg/m^2^	4	21
Cyclophosphamide (C)	600 mg/m^2^		
CMF			
Cyclophosphamide (C)	600 mg/m^2^		
Methotrexate (M)	40 mg/m^2^	6–9	21
5-fluorouracil (F)	600 mg/m^2^		
T + CDDP			
Docetaxel (T)	75 mg/m^2^		
Cisplatin (CDDP), or	50 mg/m^2^	4	21
Carboplatin	target AUC (5 mg/ml/min) x (Ccr + 25)		
TCH			
Docetaxel (T)	75 mg/m^2^		
Cisplatin (CDDP), or	50 mg/m^2^		
Carboplatin	Target AUC (5 mg/ml/min) × (Ccr + 25)	6	21
Trastuzumab (H)	Loading: 8 mg/kg Maintain: 6 mg/kg		

*AUC* area under the curve, *Ccr* creatinine clearance rate

**Table 3:** Chemotherapy regimens of the breast cancer patients without CKD (*n* = 42)

Subtype/Stage	No. of patients	Regimen	Reduction of dose or cycles	RDI
Luminal A	23		4	
I	8	CMF × 9	−	
IIA/B	2	CMF × 9	−	
	2	CEF × 6	−	
	3	CEF × 4, T × 4	−	
IIIA/B/C	4	CE (70) F × 4	+	38.9%
	1	CEF × 6	−	
	1	CEF × 6, T + CDDP × 4	−	
	1	E + T × 3, T + CDDP × 4	−	
	1	E + T × 3, CEF × 2, T × 2	−	
Luminal B	5		0	
I	2	CEF × 6, H for one year	−	
IIB	1	CEF × 4, T × 4	−	
IIIA	1	T + CDDP + H × 4, H for one year	−	
	1	E + T × 3, CEF × 6, TH × 4, H for one year	−	
Her-2-enriched	6		2	
I	1	CMF × 9	+	50.0%
	1	CEF × 6, TH × 4, H for one year	−	
IIA/B	1	CMF × 3	+	16.7%
	1	CEF × 6, H for one year	−	
IIIA/B	2	CEF × 4, TH × 4, H for one year	−	
TNBC	8		0	
I	3	CMF × 9	−	
IIA/B	2	CEF × 6	−	
	1	AC × 4, T + CDDP × 4	−	
IIIA/B/C	2	CEF × 4, T + CDDP × 4	−	

*CKD* chronic kidney disease, *RDI* relative dose intensity, *TNBC* triple-negative breast cancer, *CMF* cyclophosphamide, methotrexate, and 5-fluorouracil (5-FU), *CEF* cyclophosphamide, epirubicin, and 5-FU, *CDDP* cisplatin, *T* docetaxel, *E* epirubicin, *H* Herceptin (trastuzumab), *AC* Adriamycin (doxorubicin) and cyclophosphamide

**Table 4:** Chemotherapy regimens in the CKD group (*n* = 20)

Subtype/Stage	No. of patients	Regimen	Reduction of dose or cycles	RDI
Luminal A	9		6	
IIA/B	1	CMF × 6	−	
	1	A + T × 4	+	90.0%
	1	CEF × 4, T × 4	−	
IIIA/B/C	1	CEF × 4, T × 4	−	
	1	CEF × 4, T × 1	+	62.5%
	1	CEF × 4	+	50.0%
	2	CE (70) F × 6	+	44.4%
	1	CE (70) F × 6, T × 4	+	77.8%
Luminal B	3		2	
I	1	CMF × 6	+	66.7%
IIIA	1	CEF × 4, TH × 4, H for one year	−	
	1	TH × 4	+	50.0%
Her-2-enriched	4		2	
I	1	CE (70) F × 6	+	77.8%
II	1	CEF × 6, TH × 6, H for one year	−	
	1	CEF × 1	+	12.5%
IIIA/B	1	TCH × 6	-	
TNBC	4		1	
IIA/B	2	CEF × 4, T + CDDP × 4	−	
IIIB/C	1	E + T × 6	−	
	1	T + CDDP × 4, CMF × 3	+	66.7%

*CKD* chronic kidney disease, *RDI* relative dose intensity, *TNBC* triple-negative breast cancer, *CMF* cyclophosphamide, methotrexate, and 5-fluorouracil (5-FU), *CEF* cyclophosphamide, epirubicin, and 5-FU, *CDDP* cisplatin, *T* Taxotere, *E* epirubicin, *H* Herceptin (trastuzumab), *AC* Adriamycin (doxorubicin) and cyclophosphamide

### Recurrent rates and survival outcome

Recurrent status and overall mortality of breast cancer patients with or without CKD are analyzed and shown in Table 5. The recurrent rate was 21% in the CKD group and 14.5% in the non-CKD group. For those with locally advanced breast cancer (stage III), the recurrent rate was 57.1% in the CKD group and 28.6% in the non-CKD group. The overall mortality rate was 46.8% in the CKD group and 14.5% in the non-CKD group, with a statistically significant difference (*P* < 0.0005). The cancer-specific mortality rate in the CKD group was 24.1 versus 88.9% in the non-CKD group (*P* = 0.001).

**Table 5:** Recurrent status and overall mortality in breast cancer patients with or without CKD (*n* = 124)

	Non-CKD	CKD	*P* value
	*N* = 62	*N* = 62	
Overall recurrence, *n* (%)	9 (14.5)	13 (21.0)	0.347
Stage 3 recurrent rate	28.6%	57.1%	0.252
Overall mortality, n (%)	9 (14.5)	29 (46.8)	< 0.0005
Related to breast cancer	88.9%	24.1%	0.001
Follow-up period (months), median (IQR)	108.0 (47.9–150.8)	73.3 (38.9–107.9)	0.005
RFS (month), median (IQR)	101.9 (43.3–151.3)	70.2 (31.6–107.9)	0.08
OS (month), median (IQR)	111.3 (47.9–158.7)	73.3 (38.9–108.1)	0.007

*CKD* chronic kidney disease, *IQR* interquartile range, *RFS* recurrence-free survival, *OS* overall survival

**Fig. 1:**
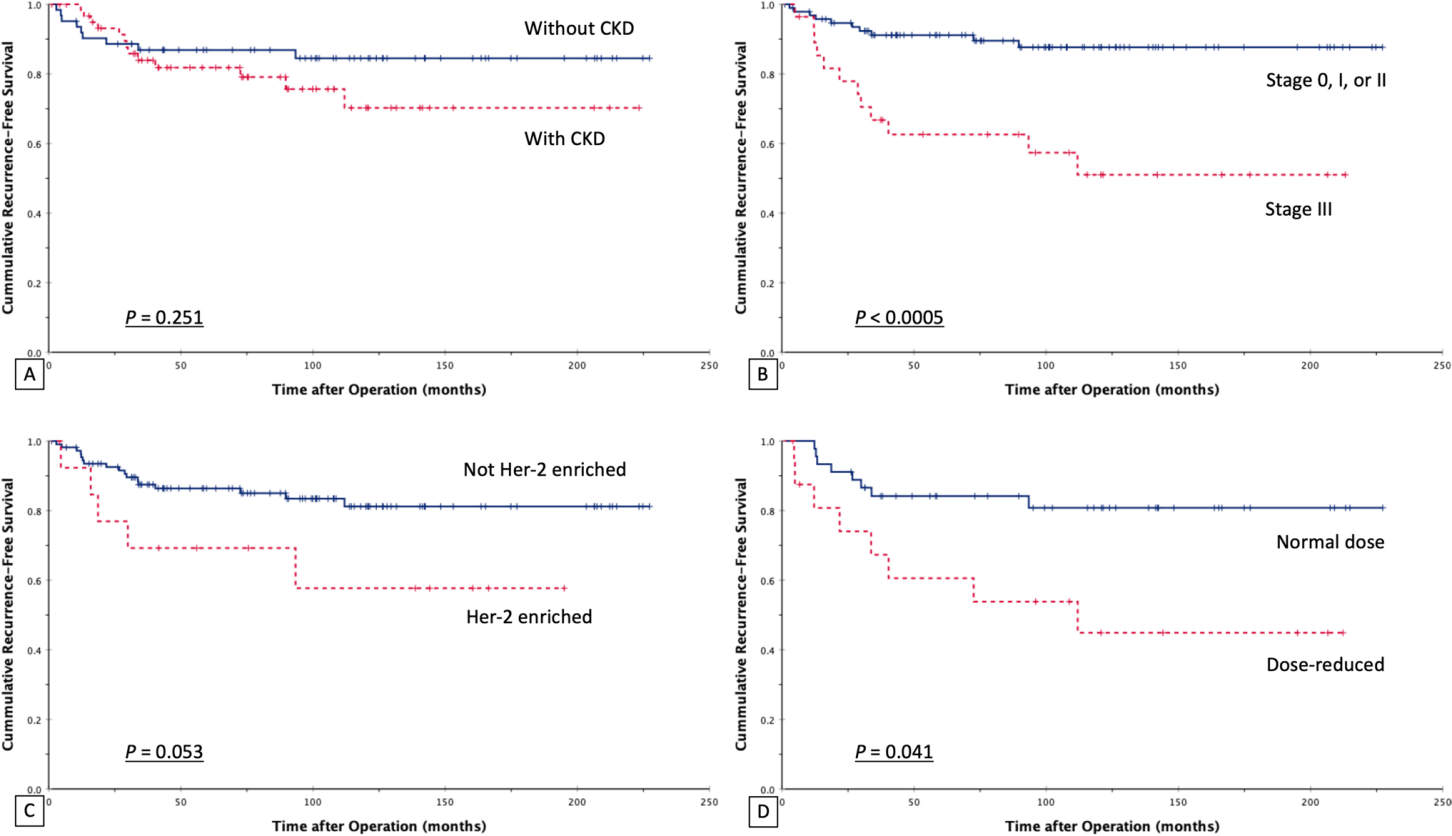
Recurrence-free survival (RFS) for breast cancer patients in relation to the possible prognostic factors. **A** CKD; **B** Stage; **C** Her-2-enriched subtype; **D** The dosage of chemotherapy. Kaplan–Meier survival curves were plotted. *CKD* chronic kidney disease

**Fig. 2:**
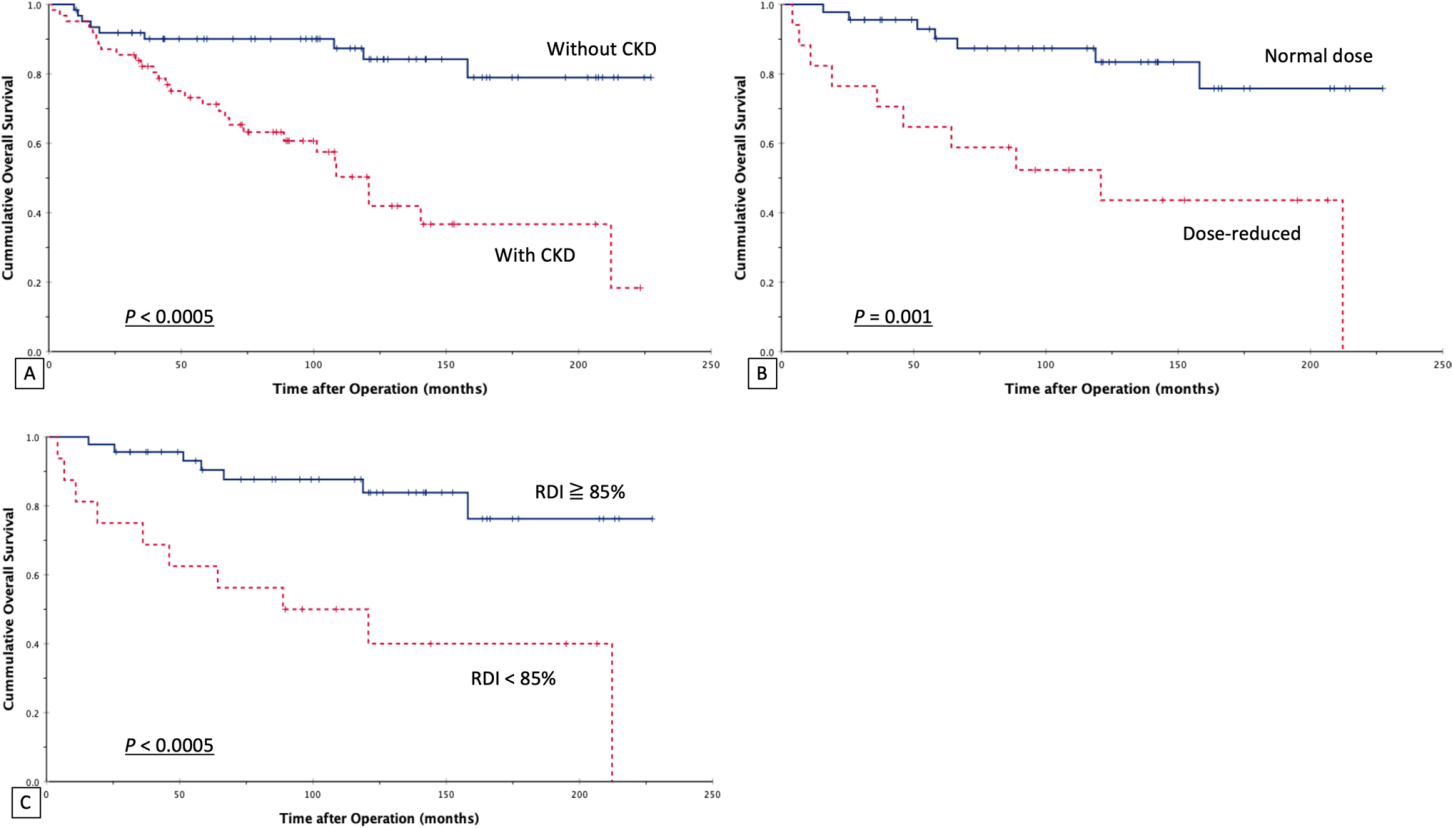
Overall survival (OS) for breast cancer patients in relation to the possible prognostic factors. **A** CKD; **B** The dosage of chemotherapy; **C** RDI. Kaplan–Meier survival curves were plotted. *CKD* chronic kidney disease, *RDI* relative dose intensity

The univariate analysis of prognostic factors affecting RFS and OS after breast cancer in those with or without late-stage CKD is provided in Table 6. 3-year, 5-year, and 10-year RFS values in the CKD group were 85.5, 81.8, and 79.0%, respectively, versus 87.1, 86.9, and 85.5% in the non-CKD group, respectively, without statistically significant difference (*P* = 0.251). 3-year, 5-year and 10-year OS values in the CKD group were 82.3, 71.2, and 59.7%, versus 91.9, 90.1, and 87.1% in the non-CKD group, respectively, with a significant difference between groups (*P* < 0.0005). The patients with stage III cancer had worse RFS (*P* < 0.0005) than did those with stage 0-II cancer. The patients who received dosage- or cycle-reduced chemotherapeutic regimens had lower rates of 5-year RFS and 5-year OS than did those receiving a standard regimen and dosage (60.6 vs 84.2%, *P* = 0.010 and 64.7 vs 90.2%, *P* = 0.001, respectively). The patients with the Her-2-enriched subtype tended to have shorter RFS (*P* = 0.053). Cox regression analysis demonstrated that dose-reduced chemotherapy was an independent negative prognostic factor for RFS. In addition, patients with CKD had a worse OS rate (Table 7). The Kaplan–Meier curves of RFS and OS based on each independent prognostic factor are shown in Fig. 1 and Fig. 2. They showed that cancer stage and dose reduction were associated with lower RFS, and CKD, does reduction, and RDI < 85% were associated with lower OS.

**Table 6:** Univariate analysis of prognostic factors affecting the RFS and OS of breast cancer patients with or without CKD

	No. of patients	5-year RFS (%)	*P* value	5-year OS (%)	*P* value
Overall	124	84.5%		80.5%	
CKD					
Yes	62	81.8%	0.251	71.2%	< 0.0005
No	62	86.9%		90.1%	
Stage					
0, I, or II	96	91.2%	< 0.0005	84.7%	0.127
III	28	62.6%		67.1%	
Subtype					
Luminal A	72	86.7%	0.227	82.5%	0.865
Luminal B	14	92.3%		90.0%	
Her-2-enriched	14	69.2%		70.7%	
Triple-negative	24	81.7%		74.6%	
Her-2-enriched subtype					
Yes	14	69.2%	0.053	70.7%	0.695
No	110	86.4%		81.8%	
Chemotherapy					
Normal dose	45	84.2%	0.041	90.2%	0.001
Dose reduction	17	60.6%		64.7%	
RDI < 85%	16	57.8%	0.097	62.5%	< 0.0005
Radiotherapy					
Yes	44	78.2%	0.318	83.0%	0.982
No	80	88.1%		79.1%	
Anti-Her-2 therapy for Her-2-positive					
Yes	10	80.0%	0.786	87.5%	0.283
No	16	79.0%		73.1%	

*RFS* recurrence-free survival, *OS* overall survival, *CKD* chronic kidney disease, *RDI* relative dose intensity

**Table 7:** Multivariate analysis of prognostic factors potentially affecting the RFS and OS of breast cancer patients with or without CKD

Factors	5-year RFS			5-year OS	
	*P*	Odds ratio (95% CI)		*P*	Odds ratio (95% CI)
General condition					
CKD	NS	–		0.002	5.211 (1.878–14.460)
Management					
Dose-reduced chemotherapy	0.015	3.383 (1.269–9.019)		NS	–

*RFS* recurrence-free survival, *OS* overall survival, *CKD* chronic kidney disease, *CI* confidence interval, *NS* not significant

### Adverse events

The most adverse events in both groups included fatigue, nausea or vomiting, hair loss, diarrhea or constipation, mucositis, poor appetite, and sensory impairment in the limbs. During the treatment period, one patient with continuous ambulatory peritoneal dialysis developed peritonitis and was temporarily switched to hemodialysis. Additionally, three patients had a decrease in cardiac ejection fraction (EF) by 43–67% compared to the original but none developed mortality. On the contrary, there were no cardiovascular events recorded in the non-CKD group.

## Discussion

Renal insufficiency is so common in patients with cancer that adjustment of the dosage or cycles of the antineoplastic drugs is often needed. However, inappropriate dosage may be related to poor outcomes [14, 15]. In the management of breast cancers, the therapeutic modalities used depend on the cancer’s stage, subtype, and accompanying risk factors. Older patients with breast cancer and severe comorbidity are at increased risk of dying from breast cancer, even after adjuvant chemotherapy is adjusted [3]. However, underlying CKD has a significant impact on the pharmacokinetics of the cytotoxic drugs used in oncological treatment. Evans et al. [18] found that impaired renal function is an independent prognostic factor, especially for those older than 70 years with renal impairment, for a poorer breast cancer-specific survival rate [19], because the reduced dosage of chemotherapeutic agents may lead to suboptimal treatment. CKD may influence the outcomes of breast cancers due to associated systemic inflammation and comorbid conditions, as well as the release of soluble mediators, such as cytokines and chemokines [20].

Bednarek et al. summarized the pharmacokinetics of the drugs currently used in treatment of breast cancers [19]. Even though some regimens may be relatively safe, in our study, only one-third of patents with late-stage CKD received chemotherapy and less than half of Her-2-enriched breast cancer patients with late-stage CKD received anti-Her-2 therapy. In clinical situations, the physicians must consider adjusting the relative safe dosage or regimens of chemotherapy; and some patients may hesitate to receive appropriate treatment based on their renal status. Therefore, we found that only 32.3% of patients in the CKD group received chemotherapeutic treatment, a rate less than half that of the non-CKD group (67.7%). Meanwhile, 55% of patients with late-stage CKD received dosage- or cycle-adjusted chemotherapy, raising concerns about the possibility of under-treatment and poorer prognosis as a result. In the current study, the CKD group had a slightly higher recurrence rate than the non-CKD group, especially those with stage III breast cancer, although the difference was not statistically significant. Although the overall mortality rate was obviously and significantly higher in the CKD group (46.8 vs 14.5%, *P* < 0.0005), the cancer-specific mortality rate was lower than in the non-CKD group (24.1 vs 88.9%). This result may indicate that the poor prognosis of patients with breast cancer and late-stage CKD is due to their underlying renal disease.

In patients with GFR 60–90 and < 60 ml/min/1.73 m^2^, the 1-year event rate of cardiotoxicity was 25 and 38%, respectively, with the best cut-off value of GFR being 78 ml/min/1.73 m^2^ [21]. Studies report that a reduced renal function represents a higher risk of developing cardiotoxicity at 12-month follow-up in patients with HER-2-positive early breast cancer treated with anti-Her-2 target therapy [15, 22]. According to our results, only 4.8% of late-stage CKD patients (totally 21.4% of patients in CKD group were Her-2-positive disease) received anti-Her-2 therapy. Anti-Her-2 treatment with trastuzumab seems to have no indication for dose adjustment, but it does imply increased risk of cardiotoxicity [19]. Although no cardio-toxic event was recorded in our study, the majority of patients in the CKD group with Her-2-positive status received no anti-Her-2 monoclonal treatment. In Taiwan, one reason why Her-2-positive subtypes (Luminal B2 and Her-2-enriched) did not receive anti-Her2 target therapy could be that the National Health Insurance only approves this therapy in those with breast cancer with positive lymph node metastasis.

NCCN guidelines call for the standard neo-adjuvant and adjuvant chemotherapy to consist of anthracycline–taxane-based regimens with or without anti-Her-2 target therapy. According to some studies related to the adjustment of chemotherapy, hormone therapy, and anti-Her-2 therapy, the reduction of chemotherapy regimens is suggested for certain agents [23, 24]. For doxorubicin, a 20% dosage reduction is recommended in patients with CKD, but none in patients on hemodialysis (HD) [23, 24]. There is no data on epirubicin in patients with HD although one study argued that a reduction in dosage should be considered [23]. However, a case report indicated the possible safety of weekly epirubicin [25]. Cyclophosphamide is excreted mostly by the kidneys, and thus, a 25% dose reduction is recommended after HD [24]. The combination regimen of epirubicin and cyclophosphamide is not recommended for patients with CKD, but a 20–25% dose reduction might be considered if needed. 5-Fluorouracil (5-FU) has a short half-life of about 16 min and only 15% of the dose is excreted unaltered into the urine; typical doses of 5-FU might be given to late-stage CKD patients [23, 24]. Cisplatin is primarily excreted through urine, and it is advisable to reduce the dose by 50–75% with prior dialysis within 30 min after chemotherapy [23, 24]. For the patients of late-stage CKD without dialysis, the application of Cisplatin is not recommended. In the study, there were two patients of triple-negative breast cancer (stage IIB and IIIC) received Docetaxel with Cisplatin for four cycles and their eGFR decreased from 28 to 22% and 29–23% respectively, without immediate requirement of dialysis. Despite no pharmacokinetic recommendations on paclitaxel and docetaxel, some case reports indicate no alterations in patients with CKD [21, 26–29]. Although the application of cyclophosphamide with a 25–30% dose reduction and methotrexate with a 75% dose reduction could be considered and administered after dialysis in patients with renal failure, the use of the cyclophosphamide, methotrexate, and 5-FU (CMF) regimen is not recommended for CKD patients with or without hemodialysis in most situations [24]. While the pharmacokinetics of trastuzumab does not change significantly in patients with HD, anti-Her-2 target therapy implies an increased risk of cardiotoxicity [22, 30, 31]. Besides, no dose reduction has been claimed for tamoxifen, anastrozole, exemestane, or fulvestrant in HD patients [23], and we did not compare the two groups by the prognosis associated with receiving hormone therapy.

Wildiers et al. indicated that breast cancer patients who achieved a defined Relative Dose Intensity (RDI) level (RDI ≥ 85%) possibly had improved Relapse-Free Survival (RFS) and Overall Survival (OS) [32]. All regimens in our cohort were listed and some patients received chemotherapy with dose reduction; the details of pharmacokinetics are difficult to analyze after dose reduction. In our study, the average RDI was 77.1% in the CKD group and 91.0% in the non-CKD group (*P* = 0.036). The 5-year RFS was lower in the patients with dose-reduced chemotherapy (*P* = 0.041), and the 5-year OS was lower in the patients with dose-reduced chemotherapy and RDI < 85% (*P* = 0.001 and < 0.0005, respectively). This result may indicate that dose-reduced chemotherapy resulted in a higher recurrence rate and poorer survival rate. However, the multivariate analyses found only one independent negative prognostic factor for 5-year OS: CKD status. Although patients with breast cancer and late-stage CKD may receive insufficient chemotherapy, which might cause a higher recurrence rate, the rate of cancer-specific mortality was not associated with dose-reduced chemotherapy in our study. Therefore, for patients with breast cancer and late-stage CKD, the OS might be related to underlying comorbidities rather than therapeutic options.

Our study has some limitations. First, the study period was extended because of limited patient numbers included in the original analysis, and discrepancies in medical care over time may have an impact on the results. Second, the follow-up period was short and the long-term effect of inappropriate management might not show up until later. Finally, this is a retrospective study with propensity score matching. Although we followed the NCCN guidelines for managements of patients with breast cancer, some alterations in care might occur as a result of physician decisions or patient socioeconomic status. Besides, the dose adjustment or reduction in patients with CKD was not standardized in the guidelines established by our institution. The decision of dose adjustment was made mainly by the physicians in charge only and thus, may influence the results. To diminish the possibility of bias on prognosis, further investigations by introducing the concepts of RDI, involving more cases, and standardizing the adjustment of dose in patients with late-stage CKD are crucial.

## Conclusions

Female breast cancer patients with late-stage CKD may receive insufficient therapeutic modalities, especially chemotherapy and anti-Her-2 therapy, in consideration of their underlying renal disease. Although the RFS rate did not differ significantly from that in the non-CKD group, the patients with breast cancer and late-stage CKD had a shorter OS time but lower breast cancer-related mortality rate, indicated that the mortality was related to underlying disease. Further prospective studies with more case numbers and standardized treatment flowchart should be conducted.

## Data Availability

The datasets generated and analyzed during the current study are available from the corresponding author on reasonable request.
